# Malignant circuits: Novel therapeutic opportunities in neuro‐oncology

**DOI:** 10.1111/jcmm.16353

**Published:** 2021-02-12

**Authors:** Kostas A. Papavassiliou, Athanasios G. Papavassiliou

**Affiliations:** ^1^ First Neurology Department Medical School Aeginition Hospital National and Kapodistrian University of Athens Athens Greece; ^2^ Department of Biological Chemistry Medical School National and Kapodistrian University of Athens Athens Greece

Recent studies discovered a surprising phenomenon in glioma and metastatic breast cancer cells that were cultured in vitro or transplanted into the brains of mice, namely, that they are capable of forming functional excitatory glutamatergic synapses with neurons and by doing so they promote their malignant behaviour.^1^, ^2^, ^3^ Specifically, when the synapses between neurons and cancer cells were activated the latter became depolarized resulting in increased cancer cell proliferation and invasiveness as well as augmented metastatic potential of cancer cells to the brain. Not only do cancer cells receive synaptic inputs from neurons and turn them into malignant signals for their own benefit but they also propagate these signals through a network of cancer cells connected via gap junctions. Moreover, there is striking evidence that brain cancer cells stimulate neurons to become more excitable via various mechanisms in order to further promote their malignancy, suggesting that neurons and cancer cells are highly associated and can impact each other in a bidirectional manner.^4^, ^5^

We are now beginning to appreciate a previously unexplored aspect of brain tumours which opens new research avenues in understanding these lethal diseases. All these new insights may be regarded as the basis of the emerging field of brain cancer neuroscience which supports that communication between neurons and cancer cells is a fundamental component of brain cancer biology, both for primary and secondary brain tumours. These findings shed some light into the puzzling nature of brain cancers, explaining, in part, why these cancers are challenging to treat and emphasizing the resilience of brain cancer cells in an environment that does not actually favour their survival. Importantly, they have major implications for the clinical treatment of brain cancers. Previously unknown drug targets are now revealed providing an opportunity to block the dynamic interplay between neurons and cancer cells in the brain at different nodes via pharmacological agents and thus potentially hamper the proliferation of brain cancers—this could lead to a paradigm shift in the treatment of neuro‐oncological patients. Focusing on the interaction of cancer cells with surrounding neurons instead of cancer cells themselves represents a unique approach to brain cancer treatment. By interrupting the malignant cues that brain cancer cells receive from their microenvironment, it could be possible to target a diverse range of brain cancer subtypes as opposed to current targeted therapies which show efficacy in only a small subset of treated patients.

Potential therapeutic targets might include structural components that brain cancer cells rely on to form synapses with neurons. Accordingly, the neuron‐derived molecule neuroligin‐3, which has been shown to up‐regulate synaptic gene expression in glioma cells,^5^ could be blocked from exerting its action through inhibiting the receptor to which neuroligin‐3 binds to; nevertheless, this receptor remains to be identified. Glutamate receptors expressed by cancer cells, particularly α‐amino‐3‐hydroxy‐5‐methyl‐4‐isoxazolepropionic acid receptor (AMPAR) in glioma cells and *N*‐methyl‐D‐aspartate receptor (NMDAR) in metastatic brain cancer cells, as well as gap junctions through which electrical signals spread among glioma cells are all promising drug targets. In addition, downstream signalling pathways that cancer cells exploit to transduce their synaptic input into malignant phenotypes represent potential therapeutic targets. Finally, targeting neuronal excitability induced by glioma is another potential strategy. By secreting glutamate via their glutamate transporter (glutamate/cystine antiporter system x_c_), glioma cells render neurons more excitable which, in turn, promotes their own growth.^6^, ^7^ Therefore, inhibiting this transporter may hinder glioma growth. Similarly, a certain mutant form of phosphatidylinositol‐4,5‐bisphosphate 3‐kinase catalytic subunit alpha (PIK3CA) expressed by glioma cells is associated with glypican 3 secretion which boosts neuronal synaptogenesis and consequently neuronal excitability,^4^ making it a promising drug target candidate.

These new approaches raise the important issue of target selectivity, that is, avoiding off‐target effects on healthy neurons since cancer cells appear to use the same synaptic processes as neurons in order to fuel their aggressiveness. This will be a major hurdle to overcome, however, it is possible that the synaptic mechanisms in cancer cells exhibit subtle differences compared with healthy neurons that could allow for selective targeting. For example, some glioma cells express a specific AMPAR that is permeable to calcium but healthy adult neurons in the brain do not express this receptor to a great extent.^1^, ^2^ Whether further differences in synapse‐related proteins between neurons and brain cancer cells exist and are pharmacologically relevant remains to be clarified.

We already have a plethora of drugs, even clinically approved ones, targeting different aspects of synaptic transmission including antiepileptic drugs. Such drugs could be repurposed and used in the future in the clinic to tackle primary brain cancers or cancers that have metastasized to the brain. Currently, there are several ongoing academic efforts as well as biotech startups that are developing promising drugs aiming at this neuron‐cancer crosstalk.

Along with these exciting therapeutic opportunities, several questions arise regarding the potential clinical use of such therapeutic interventions. Were these therapies to reach the neuro‐oncology clinic, which patients should they be administered to? Given the inherent heterogeneity of brain cancers, we need to define molecular profiles that can be used as predictive biomarkers of clinical relevance in order to identify patients who are going to benefit the most from these new therapies. Relatedly, discovering response biomarkers will be key to monitor the efficacy of such therapies. In terms of how these therapies should be administered, will it be beneficial to combine them with standard treatments, with each other or use them as monotherapy? Since these potential therapies focus on the interaction of brain cancer cells with their microenvironment, should we combine them with agents that inhibit other microenvironmental factors promoting malignant behaviour such as immunotherapy and anti‐angiogenic therapy? These questions can only be addressed through well designed randomized controlled clinical trials.

Further research on the field of brain cancer neuroscience is required to elucidate the molecular details of the neuron‐brain cancer cell interaction and its clinical significance in cancer initiation and progression. Future studies might reveal that this interaction also exists in other primary and secondary brain cancers and perhaps that other neurotransmitter systems besides glutamatergic signalling are also involved in this crosstalk. Additionally, developing imaging modalities that could potentially assess the activity of neuron‐to‐cancer cell communication would certainly assist in unravelling important features of brain cancer biology and developing new therapies. Breaking the malignant interplay between neurons and brain cancer cells is now rising as a novel therapeutic opportunity and could lead to improved outcomes that are urgently needed for neuro‐oncological patients.

## CONFLICT OF INTEREST

The authors declare that there is no conflict of interest.

## AUTHOR CONTRIBUTIONS

**Kostas A. Papavassiliou:** Conceptualization (equal); Writing‐original draft (lead). **Athanasios G. Papavassiliou:** Conceptualization (equal); Writing‐review & editing (lead).
